# Implementation strategies to integrate academically qualified nurses in German innovator hospitals

**DOI:** 10.1093/eurpub/ckac129.448

**Published:** 2022-10-25

**Authors:** J Köppen, J Kleine, CB Maier

**Affiliations:** Department of Healthcare Management, Technische Universität Berlin, Berlin, Germany

## Abstract

**Background:**

In Germany, the number of Bachelor and Master programs for nurses has increased significantly over the past 20 years but the recommended target of 10%-20% of academically qualified nurses has not yet been reached. In university hospitals, it was 3% in 2018. Major challenges for hospital managers are to attract and retain academically qualified nurses in clinical practice, with some hospitals doing better than others.

**Methods:**

In 2020, semi-structured interviews (n = 18) were conducted with chief nursing officers, nurse managers, nurses, and physicians working in five innovator hospitals, characterised by a high willingness to change the work environment, improve quality of care, and increase the number of academically qualified nurses. The interviews were recorded, transcribed, and analysed using Atlas.ti. Mayring's qualitative content analysis method was applied.

**Results:**

Recruiting, retaining, and integrating academically qualified nurses varied in the five hospitals studied but all provided time and/or financial support for nursing students. Those with a longer tradition of attracting academically qualified nurses were following a hospital-wide strategy. CNOs or other leaders motivated nurses to enrol in a degree program, provided close support for clinical projects (e.g., on the ward) and when starting a career (e.g., coaching), and fostered individual career planning. Specialised tasks for nurses with a master's degree were leadership roles or specialised patient care. Taking over additional clinical or scientific activities according to their qualification was seen as beneficial to integrate the nurses. Barriers were the limited acceptance of graduated nurses by nurses with a vocational training, low staffing levels, and limited political support.

**Conclusions:**

Recruiting and retaining graduated nurses takes efforts by hospitals in the current situation of a nation-wide nursing shortage. A hospital-wide approach can be a way to overcome this challenge.

**Speakers/Panelist:**

**Inge Rinzema**

V&VN VS, Dutch Association of Nurse Practitioners, Utrecht, Netherlands

**Johanna Heikkilä**

JAMK University, Jyväskylä, Finland

**Sabine Valenta**

Department of Nursing Science, University of Basel, Basel, Switzerland

